# Validation of a B cell acute lymphoblastic leukemia xenograft rat model for integrated efficacy, pharmacokinetics and safety of CD19 CAR-T

**DOI:** 10.1007/s43188-026-00343-1

**Published:** 2026-03-14

**Authors:** Joo-Il Kim, Mi-Young Park, Euna Kwon, Tomoji Mashimo, Hyoung Jin Kang, Byeong-Cheol Kang

**Affiliations:** 1 Department of Experimental Animal Research, Biomedical Research Institute, Seoul National University Hospital, Seoul, Korea; 2 Department of Pediatrics, Seoul National University College of Medicine, Seoul National University Cancer Research Institute, Seoul National University Children’s Hospital, Seoul, Korea; 3 Division of Animal Genetics, Laboratory Animal Research Center, Institute of Medical Science, The University of Tokyo, Minato-Ku, Tokyo, Japan; 4 Graduate School of Translational Medicine, Seoul National University College of Medicine, 101 Daehak-Ro, Jongno-Gu Seoul, 03080 Korea; 5 Institue of Laboratory Animal Resources, Seoul National University, Seoul, Korea

**Keywords:** B-ALL rat model, CD19 CAR-T, Toxicity assessment, Efficacy test, PK study

## Abstract

In preclinical studies of B cell acute lymphoblastic leukemia (B-ALL), mouse models have limitations related to their small body size, which restricts serial sampling required for integrated assessment of efficacy, safety, and pharmacokinetics (PK) assessments. To overcome these limitations, we developed a B-ALL xenograft rat model as an integrated platform for the preclinical evaluation of cell therapies and validated this platform by testing its ability to simultaneously assess efficacy, PK, and safety using a CD19 CAR-T standard. The B-ALL rat model was established using NALM-6 (human B-ALL cell line) cells, followed by intravenous injection of CD19 CAR-T three days later at doses of 1.0 (low) and 2.0 × 10⁸ cells/kg (high). B-ALL xenograft rat model successfully differentiated dose-dependent responses, demonstrating significant tumor regression without adverse effects in high-dose group, whereas low-dose group showed no efficacy. Kinetic analysis revealed peak expansion of CAR-T cells in high-dose group 3 weeks after injection, while low-dose group displayed no significant expansion. Crucially, this study demonstrates the utility of the model as an integrated PK-Efficacy evaluation platform with exploratory safety monitoring. We identified 2.0 × 10⁸ cells/kg as an effective therapeutic dose with a favorable safety profile, showing complete remission without evident toxicity under the tested conditions. This study is the first to validate a B-ALL xenograft rat model capable of simultaneously analyzing efficacy, safety, and biodistribution. By utilizing rats, a species widely accepted in toxicology, this disease-relevant model provides a robust platform to support the preclinical assessment of next-generation cell-based therapies.

**Supplementary Information:**

The online version contains supplementary material available at https://doi.org/10.1007/s43188-026-00343-1.

## Introduction

In preclinical studies, animal models are instrumental in developing advanced therapies, providing a platform for detailed assessments of efficacy, safety, and pharmacokinetics (PK) [1, 2]. In recent years, there has been a growing emphasis on the use of appropriate disease animal models in preclinical safety assessments of biopharmaceuticals [3, 4]. Regulatory guidelines increasingly recommend conducting toxicity studies in animal models that closely mimic the disease context, as this enhances the translational relevance of safety data [5–7]. Thus, the selection and validation of a suitable animal model that can integrate these assessments has become a critical step in the preclinical evaluation process [8, 9].

In particular, cancer xenograft mouse models, such as the nonobese diabetic/severe combined immunodeficiency (NOD/SCID) and NSG strains, are particularly prevalent in the safety study of anticancer biopharmaceuticals in preclinical studies. [10, 11]. Among these, the NALM-6 xenograft mouse model has seen substantial use in preclinical B-ALL studies due to its robust support for human leukemia cell engraftment [12]. However, mouse models possess critical limitations for regulatory toxicology, such as their small size impeding the serial blood sampling required for robust PK and safety biomarker analysis [13]. This, combined with challenges like the rapid development of graft-versus-host disease (GVHD), compromises long-term safety assessments of human-derived cell-based therapies [14, 15]. Consequently, mouse models are often unsuitable for generating the comprehensive, integrated data package required for regulatory safety submissions [16].

Rats, a foundational species in toxicology, offer distinct advantages that directly address these limitations [17, 18]. Their larger body size facilitates the serial blood sampling necessary for simultaneous PK, pharmacodynamic, and safety monitoring, while their longer lifespan permits long-term safety assessments [15]. Moreover, the vast repository of background toxicology data available for rats enhances the reliability and interpretation of safety evaluations [19]. Despite these benefits, the application of xenograft rat models for the integrated evaluation of cell therapies lacks formal validation. We previously reported the initial establishment of a B-ALL xenograft model using immunodeficient F344-*Il2rg/Rag2*^*em1Iexas*^ rats [20]. However, that work did not comprehensively validate its utility as an integrated platform for regulatory toxicology.

Therefore, the aim of this study was to formally validate this B-ALL xenograft rat model as an integrated platform for the clinical evaluation of cell therapies. To achieve this, we used a CD19 CAR-T reference standard as a benchmark tool to challenge the platform. We assessed the model's ability to simultaneously generate data on efficacy, safety, and PK assessment following a single-dose injection. Over a 5-week observation period, we conducted a feasibility study for an integrated PK-Efficacy evaluation platform with exploratory safety monitoring, monitoring clinical signs, body weights, food and water intake, and performing blood, serum biochemistry, coagulation tests, macroscopic observation, organ weight measurements, and histopathological analysis. A key objective was to demonstrate the model's utility as a versatile platform by identifying an effective therapeutic dose with a favorable safety profile. This study validates a novel rat xenograft platform capable of providing a simultaneous assessment of efficacy, pharmacokinetics, and safety for cell therapies. By bridging the gap between mechanistic efficacy models and preclinical safety assessments, this validated platform may serve as a robust screening tool to optimize dose and safety parameters prior to resource-intensive non-human primate studies.

## Materials and methods

### Experimental animals

F344-*Il2rg/Rag2*^em1Iexas^ rats were bred and maintained at the animal facility of Seoul National University Hospital (SNUH). The founding colony was originally established with animals purchased from the National Bio Resource Project-Rat at the University of Tokyo, Japan. Animals were acclimatized for at least 5 days in the experimental room prior to experimentation and housed under a 12/12-h light and dark cycle, at a temperature of 22 ± 2℃, and 40 − 60% humidity, with access to food (Teklad Certified Irradiated Global 18% Protein Rodent Diet 2918C, Envigo, USA) and sterilized water freely. To ensure statistical reliability while adhering to the 3R principles, the sample size was determined to be 6 animals per group. Rats were injected 5.0 × 10^6^ cells/kg of Luc-NALM-6 cells via the tail vein. Post-injection, animals were monitored for clinical signs daily, and body weight was measured twice a week. The observation period was set to 5 weeks, representing the maximal feasible duration determined by the mortality of the untreated disease control group in preliminary experiments, while being sufficient to cover acute to sub-acute toxicity. Animals were euthanized upon a decrease in body weight of more than 20% or the onset of hind limb paralysis due to leukemic burden under deep anesthesia with isoflurane. No animals or data points were excluded from the analysis in any experimental group.

### Tumor cell lines

Luciferase-expressing NALM-6 cells were kindly provided by Dr. Kyongho Choi (Seoul National University, College of Medicine). These cells were originally generated by transducing parental NALM-6 cells (ATCC) with a lentiviral vector encoding firefly luciferase and cultured in RPMI-1640 media (Welgene, Republic of Korea) supplemented with 10% fetal bovine serum (FBS, Gibco, USA) and 1% penicillin/streptomycin (Pen-strep, Gibco, USA.). Cell viability was assessed using trypan blue and an automated cell counter (Luna II, Logos Biosystems, Republic of Korea).

### CD19 CAR-T reference material and characterization

The CD19 CAR-T cells used as a reference standard for this platform validation study were produced in accordance with previously published methods [12]. All procedures involving human participants were approved by the Institutional Review Board (IRB) of Seoul National University Hospital (SNUH-IRB; 1606–033-768). Briefly, peripheral blood mononuclear cells (PBMCs) from healthy volunteers were transduced with a lentiviral vector (LTG1563; Lentigen) encoding a CAR construct (FMC63 scFv, 4-1BB costimulatory domain, CD3-zeta signaling domain). T cells were activated, transduced, and expanded using the automated CliniMACS Prodigy system (Miltenyi Biotec) as described previously [12]. The transduction efficiency of the final CAR-T cell product was confirmed by flow cytometry using a biotin-labeled anti-CD19 antibody and a PE-conjugated anti-biotin antibody (FACS-LSR II; BD) prior to injection.

### Efficacy assessment in the B-ALL rat model

To validate the B-ALL xenograft rat model's capacity for integrated assessment, a study was conducted using five experimental groups. All rats were randomly divided into five groups based on their body weights (6 per sex per group, total 60 rats). Naive control group received 10 ml/kg of saline without a tumor xenograft, while the other four groups were injected with 5.0 × 10^6^ cells/kg of NALM6 via the tail vein. Three days later, these groups received intravenous injections of either saline (saline control), untransduced T cells (mock T control), or 1.0 (low-dose) or 2.0 × 10^8^ cells/kg (high-dose) of CD19 CAR-T cells (Table 1). The model's ability to track efficacy was evaluated using an in vivo imaging system (Lumina II, PerkinElmer, USA) on days 7, 14, 18, 21, 25, 28, 32, and 35 after injection. Rats were anesthetized with 2–3% isoflurane gas and imaged 20 min after receiving 150 mg/kg D-Luciferin (Promega, USA).

**Table 1:** Experimental design of efficacy evaluation in B-ALL xenograft rats

Group	NALM-6 (cells/kg)	Test Item	Dosage (cells/kg)	Sex	No. of animals	Route of Administration
Control	0	Saline	0	Male	6	I.V
				Female	6	
Saline Control	5.0 × 10^6^	Saline	0	Male	6	I.V
				Female	6	
Mock T Control		Mock T	2.0 × 10^8^	Male	6	I.V
				Female	6	
Low		CD19 CAR-T	1.0 × 10^8^	Male	6	I.V
				Female	6	
High			2.0 × 10^8^	Male	6	I.V
				Female	6	

I.V., Intravenous

### Five-week toxicity assessment in the B-ALL rat model

During the experiment, body weights, food, and water consumption were measured twice a week, and clinical signs were observed daily. Rats were euthanized under 2–3% isoflurane 5 weeks after CAR-T cell injection. Whole blood was collected from the vena cava for hematologic analysis (ADVIA 2120i, Siemens Healthcare, Tarrytown, NY, USA). Hematology parameters measured included white blood cell (WBC), red blood cell (RBC), platelet (PLT), hematocrit (HCT), reticulocytes (RETIC), hemoglobin (HGB), mean corpuscular hemoglobin (MCH), mean corpuscular volume (MCV), mean corpuscular hemoglobin concentration (MCHC) and differential WBC. To assess serum chemistry changes during the study, serum was analyzed using an automatic chemistry analyzer (Hitachi 7070, Hitachi, Tokyo, Japan). Serum biochemistry parameters included blood urea nitrogen (BUN), low density lipoprotein (LDL), high density lipoprotein (HDL), total cholesterol (TC), total protein (TP), albumin, total bilirubin (TB), alkaline phosphatase (ALP), aspartate transaminase (AST), alanine transaminase (ALT), creatinine, triglyceride, glucose, potassium, chlorine, sodium, calcium, and phosphorus.

Five weeks after CAR-T cell injection, animals were euthanized and organs were macroscopically examined. Liver, spleen, kidney, brain, lung, heart, adrenal glands, and testes were weighed and fixed in 10% neutral buffered formalin, while the eyes and testes were preserved in Davidson's and Bouin’s solution, respectively. The fixed organs were embedded in paraffin wax, sectioned into 4–6 μm thick pieces, and stained with hematoxylin and eosin (H&E). Histopathological analyses were conducted on the liver, spleen, pancreas, kidney, brain, lung, heart, adrenal glands, gastrointestinal tract, femur, sternum, nasal cavity, ovary, and testes. All slides were subsequently examined by a board-certified toxicologic pathologist who was blinded to group assignments.

### Pharmacokinetic (PK) analysis in the B-ALL rat model

To validate the platform's suitability for kinetic analysis via serial sampling, the persistence of CD19 CAR-T cells was monitored in peripheral blood of leukemia xenograft rats, blood was collected from tail veins weekly after CD19 CAR-T cell injection (*n* = 3 / sex / group). Whole blood was collected in K_2_EDTA containing tubes (BD Microtainer, USA), from which 200 μL of the buffy coat was isolated and stored at − 80℃ in a deep freezer until DNA extraction. DNA was extracted using DNeasy Blood & Tissue Kits (QIAGEN, Germany) as per the manufacturer’s instructions, and its concentration was determined using a microplate reader (Epoch, BioTek, USA). CD19 CAR specific primers and probes were provided by Bosung Scientific Co. (Seoul, Republic of Korea) with the following sequences: Probe (FAM-ACT TGG AAC AAG AGG ACA TCG CCA-QSY), forward primer (5’-AAA CTG CTG ATC TAC CAT AC-3’), and reverse primer (5’-TCC TTG TTG ACA GAA GTA AG-3’). All reactions were performed using a ViiA7 Real-Time PCR System (Applied Biosystems, USA).

### Ethical statement

All experiments received approval from the Institutional Animal Care and Use Committee at Seoul National University Hospital (SNUH-IACUC; 20–0170) and this study was conducted as a non-GLP study. The animals were housed in a facility accredited by AAALAC International (#001169), adhering to the 8th edition of the Guide for the Care and Use of Laboratory Animals, the National Research Council, and the ARRIVE guidelines. Information regarding animal group allocation was accessible solely to the researcher overseeing the randomization process. During the experiments, researchers administering injections and recording data were blinded to group assignments, and those assessing outcomes were similarly kept unaware of the group allocations to minimize bias. The group allocation was disclosed only after the analysis of data was concluded.

### Statistical analysis

Data are presented as mean ± standard deviation (SD). Statistical analyses were conducted using one-way ANOVA, followed by Duncan's multiple range test with SPSS software version 22.0 (SPSS Inc., Chicago, IL, USA). P-values less than 0.05 were considered statistically significant.

## Results

### Characterization of CD19 CAR-T reference standard

**Fig. 1:**
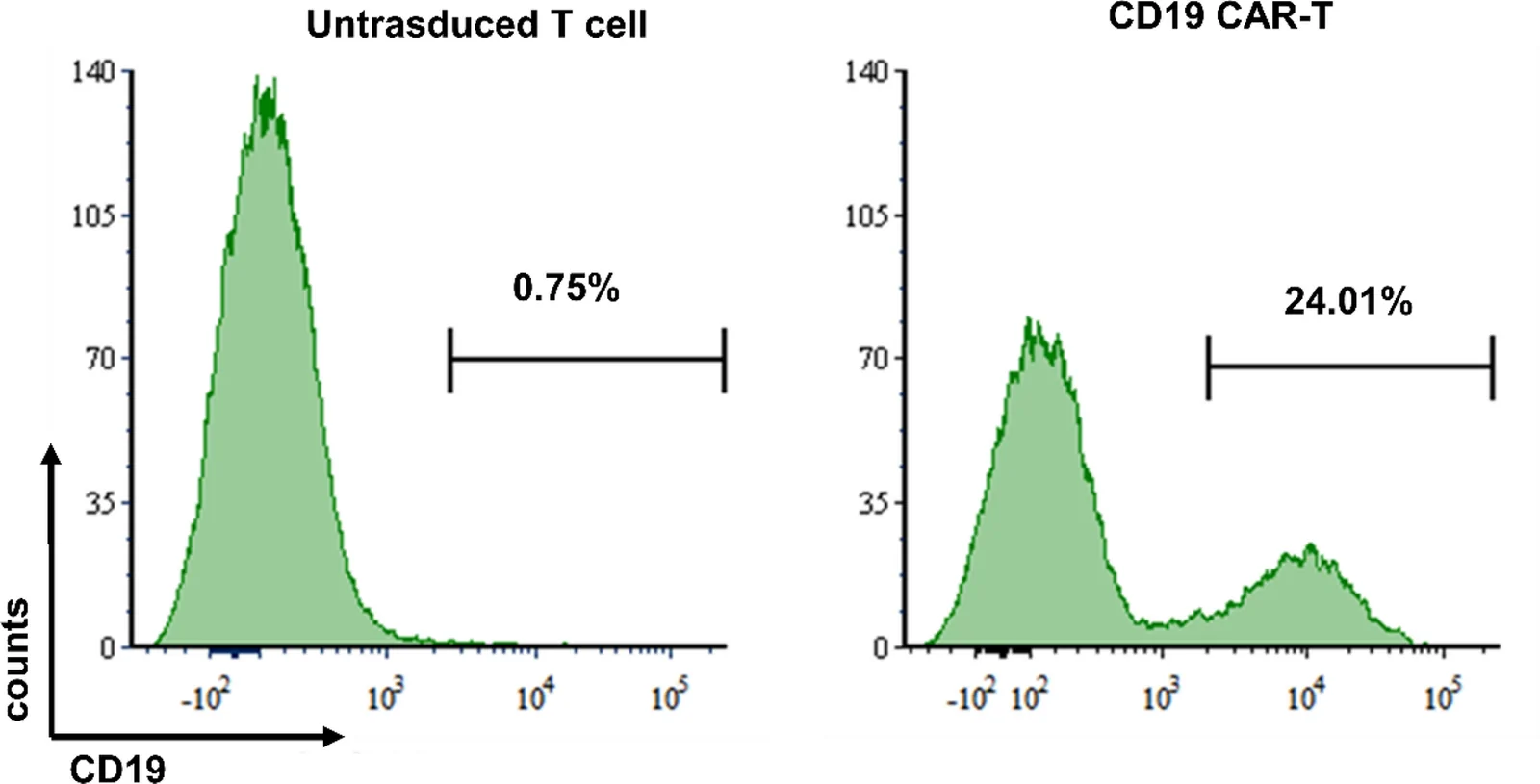
Transduction efficiency of CD19 CAR-T cells using flow cytometry CAR expression was detected by its binding to Biotin-CD19 and secondary stain for PE-anti-Biotin. Transduction efficiency was 24.01% in CD19 CAR-T cells, and 0.75% in untransduced (mock) T cells, which served as a negative control

As a prerequisite for its use as a validation tool, the CD19 CAR-T reference standard was characterized. The transduction efficiency of the targeted CD19 CAR was assessed, and its surface expression on T cells was determined to be 24.01%. In contrast, the surface expression on untransduced (mock) T cells, which acted as a negative control, was 0.75% (Fig. 1).

### Validation of the model's capacity to assess anti-leukemic efficacy

**Fig. 2:**
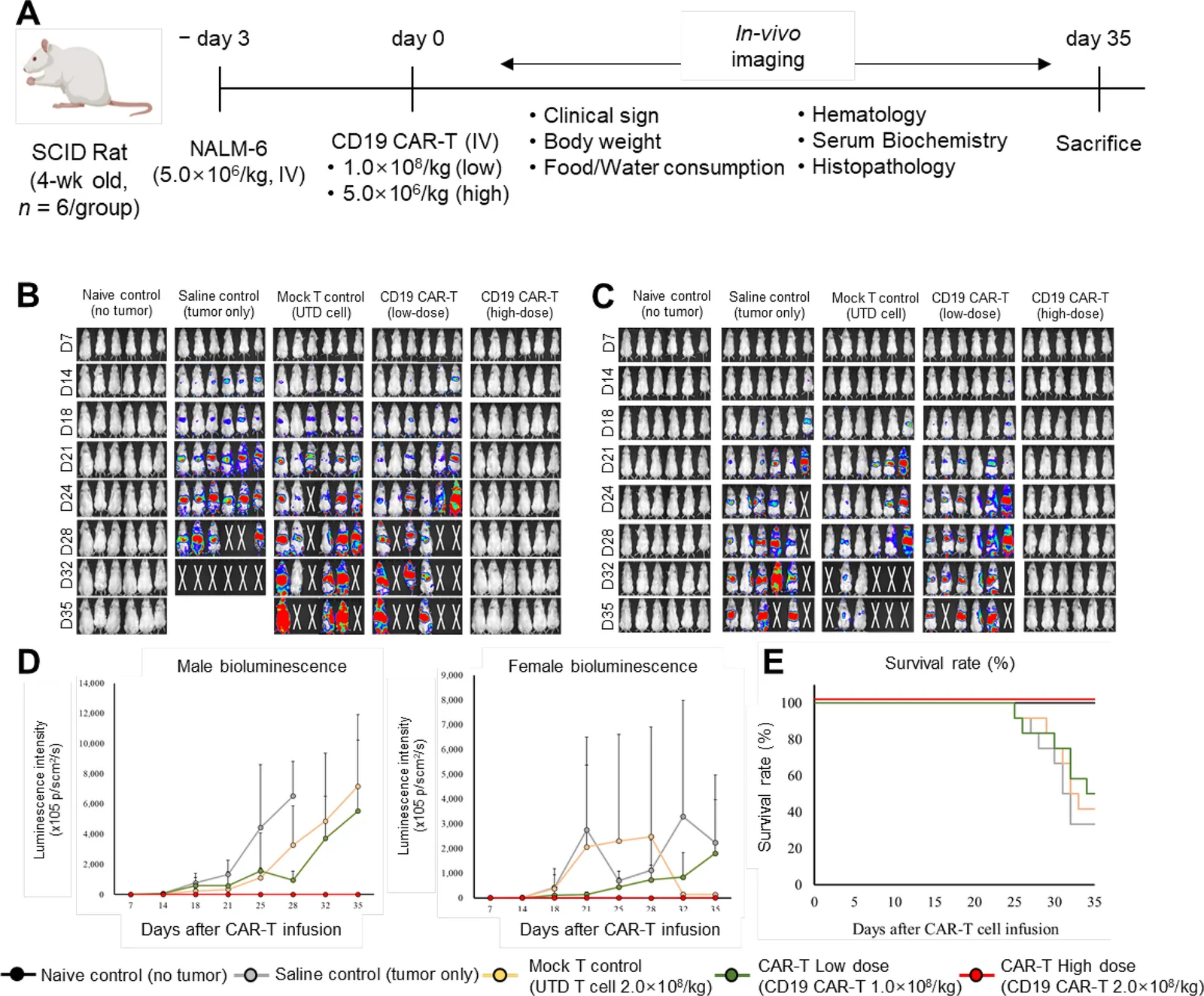
Anti-leukemic efficacy of CD19 CAR-T cells in NALM6 xenograft SCID rats **a** Schematic diagram evaluates the efficacy and toxicity of CD19 CAR-T cells in a B-ALL xenograft rat model. In both **b** male and **c** female rats, tumor progression was observed in saline, mock T control, and low-dose groups, while high-dose CD19 CAR-T cell group exhibited substantial efficacy as **d** quantified by the average bioluminescence. Although absolute tumor signals varied between sexes, the therapeutic efficacy was consistent in both males and females. **e** The survival rate was significantly higher in high-dose CD19 CAR-T treated group compared to saline, mock T control, and low-dose CD19 CAR-T groups, which required euthanasia between days 25 and 35 due to tumor burden

To validate the B-ALL rat model's capacity to assess therapeutic efficacy, its response to the CD19 CAR-T reference standard was evaluated through in vivo imaging on days 7, 14, 18, 21, 25, 28, 32, and 35 following CAR-T cell injection (Fig. 2A). The model successfully differentiated dose-dependent responses showing significant efficacy in high dose group (2.0 × 10^8^ cells/kg), in contrast to the progression of leukemia observed in saline control, mock T control, and low dose groups (1.0 × 10^8^ cells/kg). This differential outcome was clearly reflected in survival rates (Fig. 2B and C). The model's utility for tracking tumor burden was further confirmed by bioluminescence, which increased in control and low dose groups, while no signal was detected in naive control and high dose groups across both sexes (Fig. 2D). Reflecting the tumor burden monitored by the platform, rats in saline control, mock T control, and low-dose groups were euthanized between days 25 and 35 post CAR-T cell injection, whereas scheduled euthanasia was carried out in naive control and high-dose groups (Fig. 2E).

### Validation of the B-ALL rat model for regulatory safety assessment of CD19 CAR-T

#### Clinical signs and functional monitoring as indicators of morbidity

**Fig. 3:**
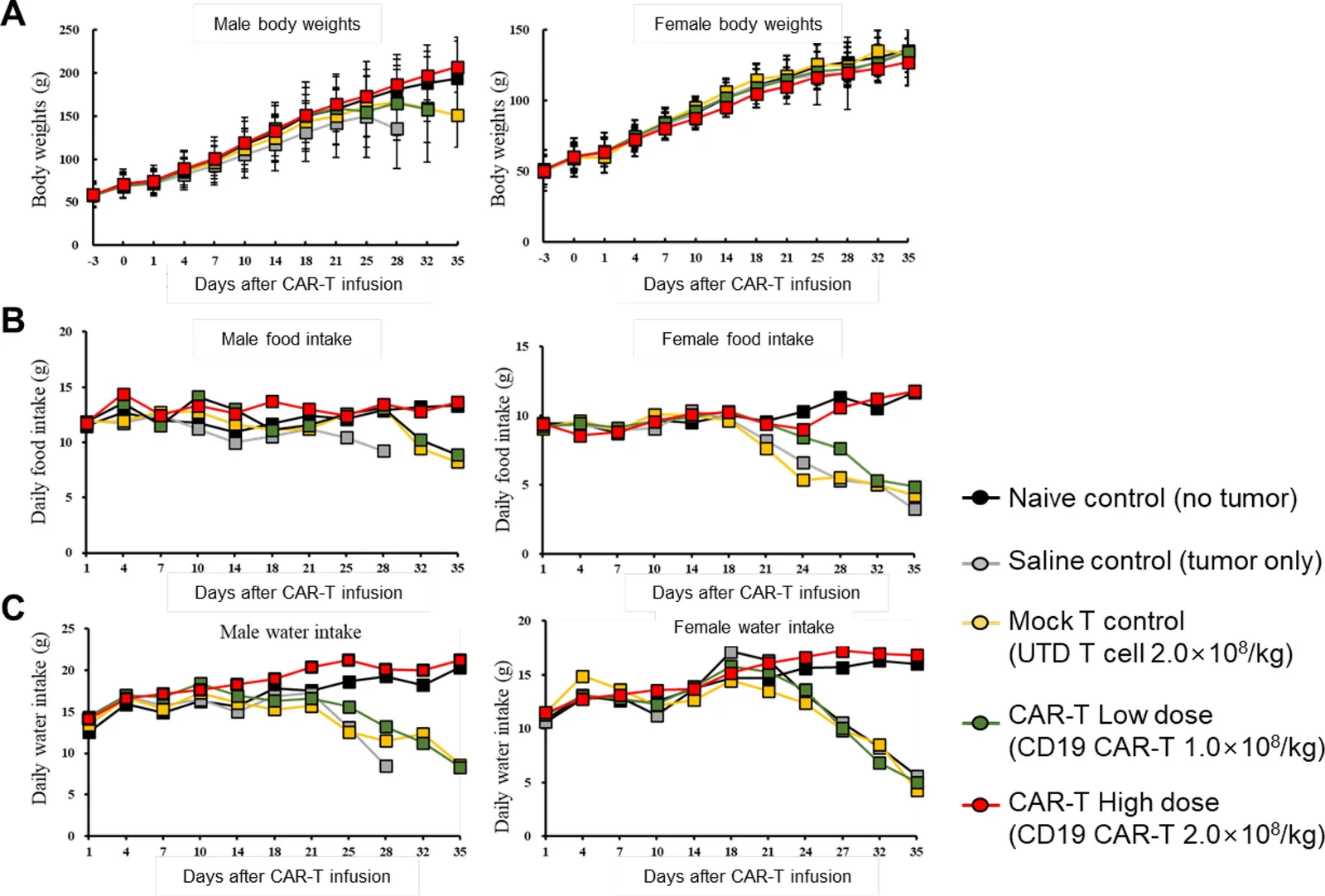
Body weights, food, and water consumption changes in NALM6 xenograft rats injected with CD19 CAR-T cells **a** Body weights decreased in saline control, mock T control, and low-dose male rats, while females exhibited no changes. Similarly, **b** food and **c** water consumption decreased in both male and female rats in saline control, mock T control, and low-dose groups. In contrast, high-dose group displayed no changes in body weight, food intake, or water intake. Data are presented as mean values

Clinical signs of morbidity began to manifest from day 25 post CAR-T cell injection. Over the course of the 35-day study, the proportion of animals requiring euthanasia due to hind limb paralysis or excessive body weight loss was as follows: saline control (male 6/6, female 3/6), mock T control (male 3/6, female 4/6), and low-dose groups (male 4/6, female 3/6). In contrast, no euthanasia was required in the naïve control and high-dose groups (Fig. 3A). In consequence, these rats were euthanized. In contrast, those in naive control and high dose CAR-T cell groups exhibited no abnormal clinical signs throughout the study in both sexes. This functional decline in the disease-progressing groups was further reflected in food and water intake, which began to decrease from day 21 in saline, mock T control, and low-dose CAR-T cell groups in both sexes, while intake in naive control and high-dose groups gradually increased (Fig. 3B and C).

#### Hematological analysis distinguishing disease-related pathology from efficacy

Hematologic analysis indicated that white blood cell (WBC) counts in saline group (male: 2.02 ± 0.62 × 10^3^ /mm^3^; *p* = 0.012, female: 1.67 ± 0.42 × 10^3^ /mm^3^, *p* = 0.013), mock T control group (male: 1.76 ± 0.85 × 10^3^ /mm^3^, *p* = 0.046; female: 1.50 ± 0.15 × 10^3^ /mm^3^, *p* = 0.032), and low-dose CAR-T groups (male: 2.15 ± 1.26, *p* = 0.006; female: 1.72 ± 0.89 × 10^3^ /mm^3^, *p* = 0.010) were elevated compared to those in naive control group (male: 0.55 ± 0.19 × 10^3^ /mm^3^; female: 0.35 ± 0.02 × 10^3^ /mm^3^). Differential WBC analysis showed an increase in the percentage of neutrophils in mock T control group (61.73 ± 7.85%,* p* = 0.034) compared to naive control (34.36 ± 6.74%) in males and a decrease in the percentage of eosinophils in saline (0.40 ± 0.19%, *p* < 0.024) and mock T control (0.46 ± 0.51%, *p* = 0.029) groups compared to naive control (2.73 ± 2.65%) in females. Furthermore, reticulocytes were increased in low-dose group of rats (male: 5.51 ± 1.28%, *p* < 0.01, female: 5.33 ± 3.13%, *p* = 0.014) compared to those in the naïve control group (male: 2.98% ± 0.89, *p* = 0.001, female: 2.19 ± 0.32%, Supplementary Table 1).

#### Serum biochemistry

Serum biochemical analysis indicated that AST levels were higher in males of saline control group (387 ± 373 IU/L, *p* = 0.027) compared to those in naive control (91 ± 5 IU/L). ALT levels showed a similar significant increase in saline control group (male: 137 ± 136 IU/L, *p* = 0.042; female: 84 ± 56 IU/L, *p* = 0.003) relative to naive control group (male: 91 ± 5 IU/L; female: 112 ± 5 IU/L). BUN levels were elevated in saline control group among females (24.25 ± 5.26 mg/dL, *p* < 0.026) when compared to naive control group (18.67 ± 1.92 mg/dL). Furthermore, TC levels in all females decreased (saline control: 57.83 ± 12.38 mg/dL, *p* = 0.031; mock T control: 56.80 ± 6.87 mg/dL, *p* = 0.028; low-dose: 49.00 ± 12.02 mg/dL, *p* = 0.001; high-dose: 54.33 ± 7.69 mg/dL, *p* = 0.008) relative to those in naive control group (74.33 ± 9.81 mg/dL). The A/G ratio decreased in both males and females of mock T control group (male: 0.70 ± 0.12, *p* = 0.019; female: 0.78 ± 0.16, *p* = 0.003) and males in low-dose group (0.68 ± 0.08, *p* = 0.009) compared to the naïve control group (male: 0.90 ± 0.11; female: 0.98 ± 0.08). Phosphorus levels were increased in males of low-dose group (9.30 ± 0.31 mg/dL, *p* = 0.014) compared to those in naive control group (6.50 ± 0.37 mg/dL, Supplementary Table 2).

#### Gross pathology and organ weight changes reflecting tumor infiltration

**Fig. 4:**
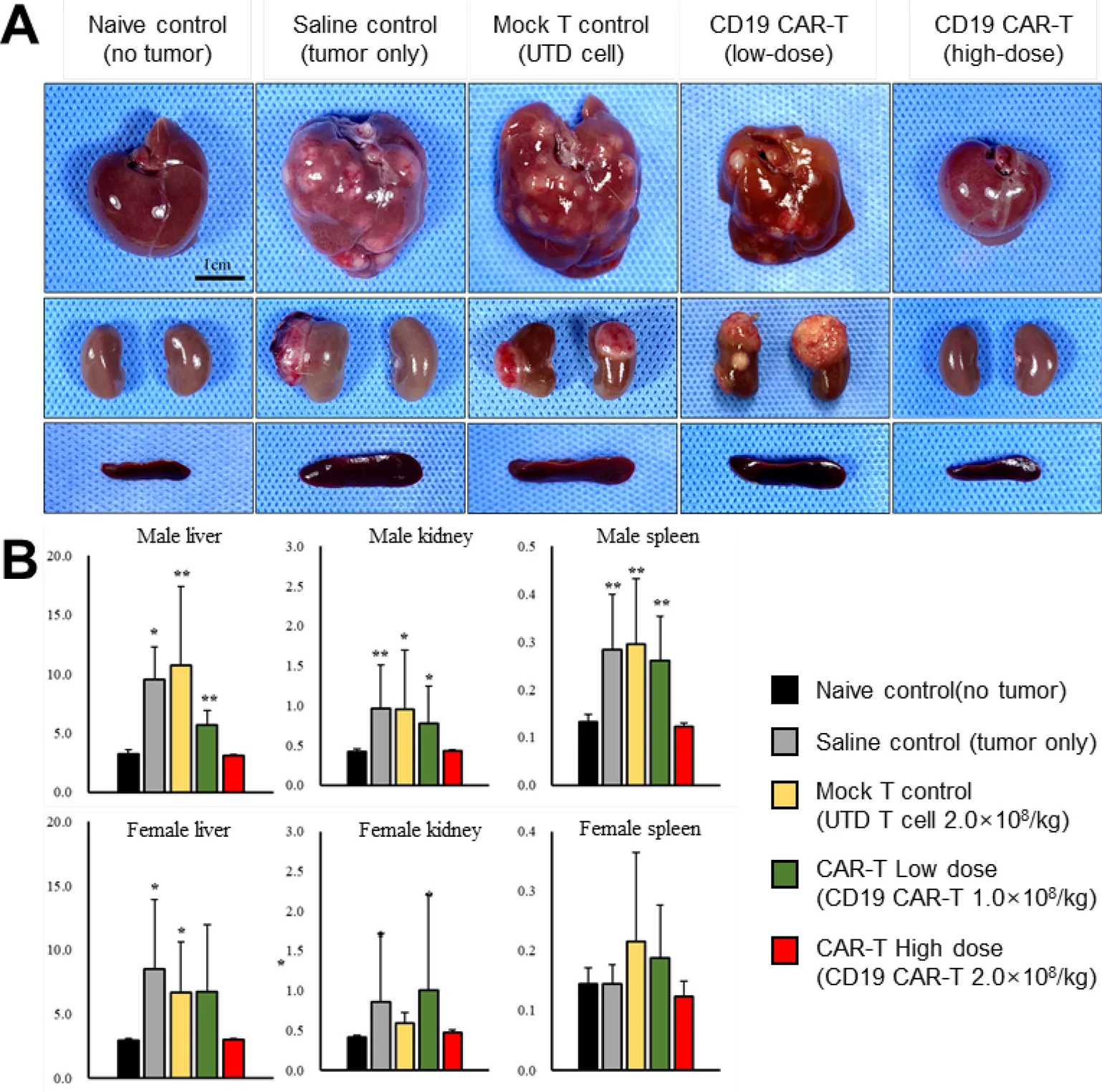
Increased liver, spleen and kidney size with white masses in NALM6 xenograft rats **a** Representative photographs show increased sizes of the liver, kidney, and spleen, with no changes observed in naive control and high-dose group. White masses in the liver and kidney were noted in saline, mock T control, and low-dose group (scale bar: 1 cm). **b** Relative liver and spleen weights in saline and mock T control groups were significantly increased compared to those in the naïve control group among males. In females, a similar significant increase was observed in liver weight; spleen and kidney weights showed an increasing trend but did not reach statistical significance. Data are presented as mean ± SD. Significance versus naive control: *, *p* < 0.05; **, *p* < 0.01

Gross pathological findings and organ weights directly correlated with the tumor burden observed in the efficacy assessment. Consistent with high tumor burden, rats in the tumor-progressing groups exhibited tumor infiltration in the liver and kidney (Fig. 4A). This infiltration corresponded with increased relative liver weights of males in saline (9.55 ± 2.75%, *p* = 0.013) and mock T control (10.73 ± 6.70%, *p* = 0.003) groups were greater compared to those of naive control group (3.21 ± 0.36%). Female weights also demonstrated a trend toward increase, although these were not statistically significant. Similarly, relative spleen weights in males of saline (0.284 ± 0.115%, *p* = 0.026) and mock T control (0.295 ± 0.137%, *p* = 0.016) groups were elevated when compared to those in naive control group (0.132 ± 0.015%, Fig. 4B and Supplementary Table 3 and 4).

#### Histopathologic analysis

**Fig. 5:**
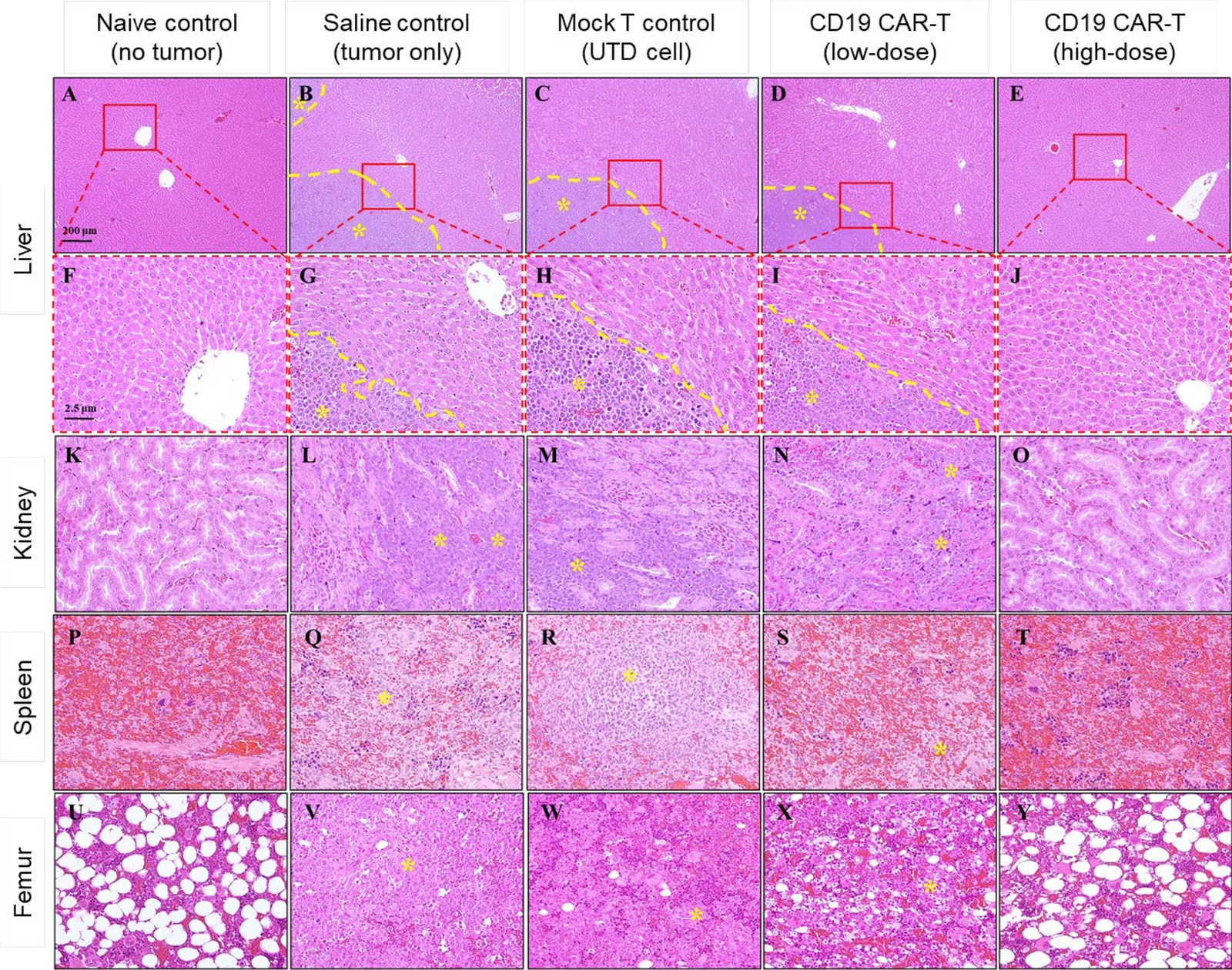
Infiltration of leukemia cells in the liver, kidney, spleen, and femur was observed only in saline, mock T control, and low-dose groups; no infiltration occurred in naive and high dose groups NALM6 cells extensively infiltrated the liver **a-j**, kidney **k–o**, spleen **p–t**, and femur **u-y**, but remained absent in naive control and high-dose groups. Dotted lines and an asterisk denote tumor cell infiltration

Histopathological analysis revealed no dose dependent lesions attributable to the administered CAR T cells. However, infiltration of tumor cells (NALM6) was observed in various organs including the liver, kidney, femur, sternum, spine, and nasal cavity in saline control, mock T, and CAR-T low dose groups (Fig. 5). In contrast, no tumor cell infiltration was detected in the naive control group and the CAR-T high dose group. (Supplementary Table 5).

### Validation of the B-ALL rat model for PK analysis

**Fig. 6:**
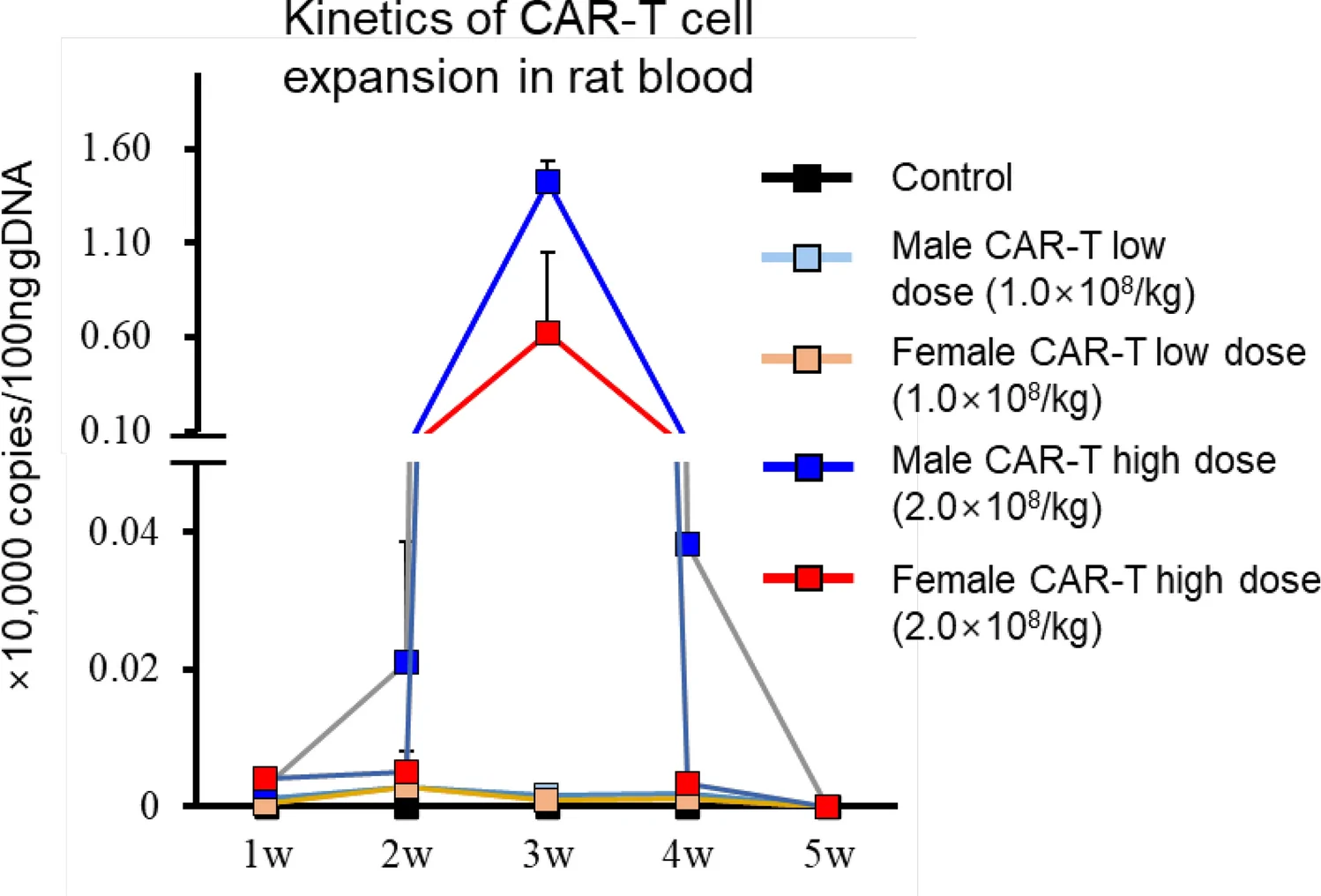
CD19 CAR-T cells in high-dose group peaked in peripheral blood at 3 weeks post-injection Whole blood was collected weekly following the CAR-T cell injections (*n* = 3 per sex, per group). Copies of CAR-specific genes detected using qPCR remained at low levels without proliferating in low-dose CAR-T group (right dotted box). In high-dose CAR-T group, they peaked at week 3 in both males and females. The right dotted box corresponds to low y-axis values. Data are presented as mean ± standard deviation

To validate the platform's suitability for PK analysis via serial sampling in rat model, whole blood was collected from the tail vein weekly. This approach successfully captured a clear, dose-dependent kinetic profile. CAR-T cells in low-dose group (1.0 × 10^8^ cells/kg) maintained low levels, never exceeding 30 copies throughout the experiment in both males and females. However, CAR gene expression levels in high-dose group (2.0 × 10^8^ cells/kg) began to escalate, peaking at 3 weeks post-injection in both males (14,278 ± 1,053 copies/100 ng gDNA) and females (6243 ± 4252 copies/100 ng gDNA) (Fig. 6).

## Discussion

The preclinical development of cell therapies, such as CAR-T, faces a significant regulatory hurdle, the requirement for an integrated data package encompassing efficacy, safety, and PK. Standard immunodeficient mouse models, while widely used, are fundamentally limited in this regard [21]. Their small body size and fragility preclude the serial, large-volume blood sampling necessary for robust PK and safety biomarker analyses, often forcing the use of separate animal cohorts and fragmented data [14, 22, 23]. Our study was designed to formally validate the B-ALL xenograft rat model we previously established as a solution to this critical gap. While our prior work demonstrated the feasibility of engraftment, the current study provides the first comprehensive validation of this model as an integrated platform for preclinical safety evaluation, utilizing rat models as a foundational species in toxicological research [20, 24, 25].

Our results demonstrate the comprehensive utility of this platform. First, the model validated its pharmacodynamic relevance by clearly differentiating the dose-dependent anti-leukemic efficacy of the CD19 CAR-T reference standard. High-dose group achieved complete tumor regression and 100% survival, whereas low-dose group failed to control the leukemia, mirroring the control groups. Second, by leveraging the rat's size for serial blood collection, we successfully bridged the gap between PK in the same individuals. We demonstrated that therapeutic efficacy was directly correlated with in vivo CAR-T expansion, as a clear PK peak at 3 weeks was observed only in the efficacious high-dose group, a level of analysis unattainable in parallel mouse studies. This 'all-or-nothing' outcome reflects the unique kinetics of CAR-T cells as a 'living drug,' where surpassing a critical effector-to-target threshold is essential to trigger robust expansion [26]. The failure of the low-dose group suggests that sub-optimal dosing leads to rapid exhaustion and clearance rather than the necessary proliferation to overcome tumor burden.

Most critically from a regulatory toxicology perspective, a key challenge in disease-relevant models is distinguishing disease-related morbidity from true drug-attributable toxicity. Our results clearly demonstrated that all adverse findings, including clinical signs, weight loss, and significant alterations in hematology, serum biochemistry, and organ weights—were exclusively confined to the non-efficacious groups (saline, mock T, and low-dose) and were directly attributable to the uncontrolled leukemia burden.

Specifically, the biochemical profile provided mechanistic insight into the disease progression. The significant elevation of AST and ALT was confined to the saline control group, reflecting extensive hepatocellular necrosis associated with terminal-stage leukemia. In contrast, the mock and low-dose groups maintained normal enzyme levels despite tumor infiltration, indicating that hepatocellular integrity was maintained in the absence of massive cellular breakdown. Notably, the low-dose group exhibited lower relative liver weights compared to controls, suggesting a partial therapeutic effect that suppressed explosive tumor growth and preserved tissue integrity.

Furthermore, the hepatic infiltration and associated organ dysfunction observed in our model are not artifacts but faithfully recapitulate the extramedullary involvement and hepatosplenomegaly frequently reported in clinical B-ALL cases, including reports of acute hepatic and renal failure [27, 28]. This confirms that the model accurately reflects the pathological characteristics of the human disease.

The hematological profiles correlated with the therapeutic outcomes. The high-dose group showed parameters indistinguishable from naïve controls, indicating the restoration of hematopoietic homeostasis following complete tumor remission. Conversely, the low-dose group exhibited significant reticulocytosis. This is likely attributable to compensatory stress erythropoiesis, a mechanism known to be activated by inflammatory signaling pathways during partial tumor clearance and active immune responses, as opposed to the marrow failure observed in the non-efficacious control groups [29, 30].

In contrast, the high-dose group, which received the highest concentration of CAR-T cells, was indistinguishable from the healthy naïve control group. This finding is significant, as it demonstrated that 2.0 × 10⁸ cells/kg serves as an effective therapeutic dose with a favorable safety profile under these conditions.

The distinct advantage of the xenograft rat model lies in its ability to integrate assessments that are typically fragmented in mouse studies. In mice, efficacy, PK, and safety assessments are often evaluated in separate cohorts, limiting the ability to correlate these critical endpoints within the same individual. Our study provides a direct solution to this fragmentation. The validated rat platform enables the simultaneous integration of efficacy, PK, and exploratory safety monitoring within a single animal. This integrative approach, which allows for the direct correlation of safety findings with drug exposure and therapeutic efficacy, enhances the comprehensive understanding of the therapeutic candidate. By generating robust data correlated across endpoints in the same individuals, this platform provides a valuable screening tool and bridging strategy to optimize dose and safety parameters prior to resource-intensive non-human primate (NHP) studies.

Limitations of this study should be considered. The CD19 CAR-T reference standard used had a transduction efficiency of 24.01%, which is lower than some clinical-grade products [31]. However, the fact that this platform still possessed the sensitivity to clearly differentiate the dose-dependent efficacy and PK of this lower-purity product arguably strengthens the validation of the model's sensitivity. Future studies should utilize this validated platform to assess cell therapies with higher transduction efficiencies or different modalities, and potentially expand its application to other indications, such as solid tumors [32]. Furthermore, the evaluation of toxicity was limited to a single efficacious dose level, as the low-dose group could not be assessed for toxicological endpoints due to the confounding effects of severe leukemic burden. While this limits the interpretation of a full dose–response relationship and margin of safety typically required in regulatory studies, the survival of the high-dose group without adverse events supports the feasibility of the model for screening therapeutic doses.

Additionally, it is important to note the limitations associated with the use of immunodeficient animals. Since these models lack a fully competent immune system, they cannot fully recapitulate the systemic inflammatory cascades seen in patients, such as Cytokine Release Syndrome (CRS) or Immune Effector Cell-Associated Neurotoxicity Syndrome (ICANS), which involve interactions between CAR-T cells and host immune component. Therefore, while our results confirm the absence of direct off-target cytotoxicity and general physiological toxicity, the potential for immune-mediated adverse events should be further assessed in immunocompetent models or clinical trials.

In conclusion, this study successfully validates a B-ALL xenograft rat model as a robust platform for the integrated preclinical evaluation of cell therapies. By enabling the simultaneous assessment of efficacy, long-term pharmacokinetics, and regulatory-style safety toxicology in a foundational toxicology species, this model provides a superior and more translatable tool. It bridges the gap between mechanistic efficacy studies and formal safety assessments, offering a new standard for generating the comprehensive data required for the regulatory approval of next-generation biopharmaceuticals.

## Supplementary Information

Below is the link to the electronic supplementary material.Supplementary file1 (DOCX 84 KB)

## Data Availability

The authors confirm that the data supporting the findings of this study are available within the article. Further raw data supporting the findings of this study are available from the corresponding author, B.C.Kang upon reasonable request.
