# Supplementary material for: Validation of a B cell acute lymphoblastic leukemia xenograft rat model for integrated efficacy, pharmacokinetics and safety of CD19 CAR-T
Source: Toxicol Res. 2026 Mar 14;42(4):535–46. doi: 10.1007/s43188-026-00343-1 (PMC13332086; doi:10.1007/s43188-026-00343-1)
Supplement: Supplementary file 1 — Supplementary file1 (DOCX 84 KB) [file 43188_2026_343_MOESM1_ESM.docx]

**Supplementary Table 1.** Hematologic values for NALM-6 xenograft rats injected with CD19 CAR-T cells

|  | Naive Control (No tumor) | | | Saline Control (Tumor only) | | | Mock T Control (Tumor + Mock T  2.0×10^8^/kg) | | | Low (Tumor + CAR-T 1.0×10^8^/kg) | | | High (Tumor + CAR-T 2.0×10^8^/kg) | | |
| --- | --- | --- | --- | --- | --- | --- | --- | --- | --- | --- | --- | --- | --- | --- | --- |
| ***n (male)*** | *6* | | | *6* | | | *6* | | | *6* | | | *6* | | |
| WBC (10^3^/mm^3^) | 0.55 | ± | 0.19 | **2.02** | **±** | **0.62^**^** | **1.76** | **±** | **0.85^*^** | **2.15** | **±** | **1.26^**^** | 0.37 | ± | 0.09 |
| RBC (10^6^/mm^3^) | 8.38 | ± | 0.39 | 7.92 | ± | 0.57 | 8.14 | ± | 0.77 | 7.76 | ± | 0.31 | 8.03 | ± | 0.14 |
| HGB (g/dl) | 14.34 | ± | 0.55 | 13.90 | ± | 1.77 | 14.57 | ± | 1.65 | 13.45 | ± | 0.52 | 14.52 | ± | 0.19 |
| HCT (%) | 43.70 | ± | 1.56 | 41.42 | ± | 4.05 | 44.05 | ± | 4.50 | 41.53 | ± | 1.16 | 43.63 | ± | 0.27 |
| PLT (10^3^/mm^3^) | 804 | ± | 195 | 683 | ± | 212 | 810 | ± | 279 | 613 | ± | 177 | 889 | ± | 63 |
| MCV (fl) | 52.22 | ± | 0.98 | 52.23 | ± | 1.94 | 54.08 | ± | 1.06 | 53.62 | ± | 1.92 | 54.33 | ± | 1.19 |
| MCH (pg) | 17.16 | ± | 0.23 | 17.50 | ± | 1.10 | 17.90 | ± | 0.64 | 17.38 | ± | 0.52 | 18.10 | ± | 0.28 |
| MCHC (g/dl) | 32.86 | ± | 0.29 | 33.47 | ± | 1.09 | 33.05 | ± | 0.58 | 32.43 | ± | 0.67 | 33.33 | ± | 0.37 |
| Neutrophils (%) | 34.36 | ± | 8.74 | 36.78 | ± | 23.04 | 61.73 | ± | 7.85 | 49.58 | ± | 15.71 | 40.23 | ± | 19.41 |
| Neutrophils (10^3^/mm^3^) | 0.18 | ± | 0.08 | 0.82 | ± | 0.65 | 1.12 | ± | 0.64 | 1.15 | ± | 0.80 | 0.15 | ± | 0.08 |
| Eosinophils (%) | 0.68 | ± | 0.44 | 0.38 | ± | 0.17 | 1.07 | ± | 1.06 | 2.30 | ± | 2.67 | 0.60 | ± | 0.55 |
| Eosinophils (10^3^/mm^3^) | 0.01 | ± | 0.02 | 0.01 | ± | 0.01 | 0.02 | ± | 0.02 | 0.03 | ± | 0.05 | 0.00 | ± | 0.00 |
| Basophils (%) | 0.54 | ± | 0.00 | 0.60 | ± | 0.33 | 0.42 | ± | 0.13 | 0.33 | ± | 0.23 | 0.55 | ± | 0.45 |
| Basophils (10^3^/mm^3^) | 0.00 | ± | 0.00 | 0.01 | ± | 0.01 | 0.01 | ± | 0.01 | 0.01 | ± | 0.01 | 0.00 | ± | 0.00 |
| Lymphocytes (%) | 54.40 | ± | 90.3 | 53.67 | ± | 21.09 | 26.40 | ± | 7.31 | 34.20 | ± | 17.19 | 49.37 | ± | 16.75 |
| Lymphocytes (10^3^/mm^3^) | 0.30 | ± | 0.12 | 1.01 | ± | 0.37 | 0.44 | ± | 0.15 | 0.69 | ± | 0.54 | 0.18 | ± | 0.08 |
| Monocytes (%) | 6.30 | ± | 3.16 | **3.38** | **±** | **0.99^*^** | 6.33 | ± | 2.48 | 4.45 | ± | 3.35 | 8.17 | ± | 4.34 |
| Monocytes (10^3^/mm^3^) | 0.04 | ± | 0.02 | 0.07 | ± | 0.03 | 0.11 | ± | 0.07 | 0.11 | ± | 0.10 | 0.03 | ± | 0.01 |
| Reticulocytes (%) | 2.98 | ± | 0.89 | 3.45 | ± | 0.60 | 2.50 | ± | 1.13 | **5.51** | **±** | **1.28^**^** | 2.69 | ± | 0.47 |
| Reticulocytes (10^3^/mm^3^) | 248.9 | ± | 72.8 | 271.0 | ± | 39.8 | 199.0 | ± | 80.2 | **425.5** | **±** | **90.7**** | 215.35 | ± | 35.63 |
| ***n (female)*** | *6* | | | *6* | | | *6* | | | *6* | | | *6* | | |
| WBC (10^3^/mm^3^) | 0.35 | ± | 0.02 | **1.67** | **±** | **0.42^*^** | **1.50** | **±** | **0.15^*^** | **1.72** | **±** | **0.89^*^** | 0.59 | ± | 0.81 |
| RBC (10^6^/mm^3^) | 8.31 | ± | 0.30 | 8.46 | ± | 0.42 | 8.02 | ± | 0.70 | 8.11 | ± | 0.84 | 8.40 | ± | 0.26 |
| HGB (g/dl) | 15.13 | ± | 0.51 | 15.48 | ± | 1.19 | 14.10 | ± | 1.11 | 14.95 | ± | 1.51 | 15.48 | ± | 0.54 |
| HCT (%) | 44.78 | ± | 1.11 | 45.26 | ± | 3.45 | 42.78 | ± | 2.71 | 44.58 | ± | 4.66 | 45.82 | ± | 1.50 |
| PLT (10^3^/mm^3^) | 873 | ± | 44 | 791 | ± | 160 | 785 | ± | 337 | 679 | ± | 237 | 758 | ± | 121 |
| MCV (fl) | 53.93 | ± | 1.03 | 53.46 | ± | 2.00 | 53.45 | ± | 2.75 | 54.97 | ± | 1.35 | 54.55 | ± | 0.74 |
| MCH (pg) | 18.20 | ± | 0.34 | 18.26 | ± | 0.75 | 17.60 | ± | 0.95 | 18.43 | ± | 0.35 | 18.43 | ± | 0.29 |
| MCHC (g/dl) | 33.75 | ± | 0.51 | 34.16 | ± | 0.27 | 32.93 | ± | 0.64 | 33.57 | ± | 0.70 | 33.80 | ± | 0.26 |
| Neutrophils (%) | 32.95 | ± | 14.93 | 35.06 | ± | 25.56 | 50.76 | ± | 23.79 | 39.84 | ± | 15.06 | 48.83 | ± | 15.97 |
| Neutrophils (10^3^/mm^3^) | 0.12 | ± | 0.06 | 0.67 | ± | 0.70 | 0.75 | ± | 0.35 | 0.75 | ± | 0.63 | 0.39 | ± | 0.69 |
| Eosinophils (%) | 2.73 | ± | 2.65 | **0.40** | **±** | **0.19^*^** | **0.46** | **±** | **0.51^*^** | 0.80 | ± | 0.62 | 0.95 | ± | 0.82 |
| Eosinophils (10^3^/mm^3^) | 0.01 | ± | 0.01 | 0.01 | ± | 0.01 | 0.01 | ± | 0.01 | 0.01 | ± | 0.02 | 0.00 | ± | 0.01 |
| Basophils (%) | 0.35 | ± | 0.29 | 0.60 | ± | 0.38 | 0.46 | ± | 0.23 | 0.62 | ± | 0.23 | 0.28 | ± | 0.29 |
| Basophils (10^3^/mm^3^) | 0.00 | ± | 0.00 | 0.01 | ± | 0.01 | 0.01 | ± | 0.01 | 0.01 | ± | 0.00 | 0.00 | ± | 0.00 |
| Lymphocytes (%) | 51.95 | ± | 16.64 | 55.06 | ± | 23.34 | 36.96 | ± | 21.43 | 46.88 | ± | 19.75 | 40.12 | ± | 12.70 |
| Lymphocytes (10^3^/mm^3^) | 0.18 | ± | 0.05 | 0.84 | ± | 0.29 | 0.56 | ± | 0.35 | 0.72 | ± | 0.45 | 0.16 | ± | 0.10 |
| Monocytes (%) | 8.03 | ± | 4.35 | 3.46 | ± | 0.46 | 8.62 | ± | 4.77 | 7.20 | ± | 2.82 | 7.70 | ± | 2.44 |
| Monocytes (10^3^/mm^3^) | 0.03 | ± | 0.01 | 0.06 | ± | 0.02 | 0.13 | ± | 0.08 | 0.13 | ± | 0.12 | 0.03 | ± | 0.02 |
| Reticulocytes (%) | 2.19 | ± | 0.32 | 3.53 | ± | 0.91 | 4.29 | ± | 3.07 | **5.33** | **±** | **3.13^*^** | 2.61 | ± | 0.51 |
| Reticulocytes (10^3^/mm^3^) | 182.0 | ± | 26.1 | 301.1 | ± | 92.2 | 333.2 | ± | 213.4 | **355.2** | **±** | **174.3*** | 219.2 | ± | 44.2 |
|  |  |  |  |  |  |  |  |  |  |  |  |  |  | | |

*, *p* < 0.05, **, *p* < 0.01.

|  | Naive Control (No tumor) | | | Saline Control (Tumor only) | | | Mock T Control (Tumor + Mock T  2.0×10^8^/kg) | | | Low (Tumor + CAR-T 1.0×10^8^/kg) | | | High (Tumor + CAR-T 2.0×10^8^/kg) | | |
| --- | --- | --- | --- | --- | --- | --- | --- | --- | --- | --- | --- | --- | --- | --- | --- |
| ***n (male)*** | *6* | | | *6* | | | *6* | | | *6* | | | *6* | | |
| BUN (mg/dl) | 17.50 | ± | 2.90 | 25.42 | ± | 3.95 | 22.94 | ± | 11.00 | 30.68 | ± | 28.60 | 15.25 | ± | 1.43 |
| TC (mg/dl) | 48.17 | ± | 8.57 | 45.50 | ± | 16.71 | 54.60 | ± | 5.90 | 47.80 | ± | 10.28 | 44.67 | ± | 8.04 |
| LDL (mg/dl) | 3.67 | ± | 1.21 | 5.50 | ± | 1.22 | **6.80** | **±** | **3.63^*^** | 5.20 | ± | 1.10 | 2.83 | ± | 1.17 |
| HDL (mg/dl) | 20.00 | ± | 3.90 | 16.50 | ± | 5.92 | 18.40 | ± | 5.37 | 19.60 | ± | 6.80 | 18.33 | ± | 2.42 |
| Total Protein (g/dl) | 5.52 | ± | 0.31 | 5.15 | ± | 0.51 | 5.12 | ± | 0.29 | 5.34 | ± | 0.25 | 5.72 | ± | 0.12 |
| Albumin (g/dl) | 2.62 | ± | 0.32 | 2.27 | ± | 0.45 | **2.12** | **±** | **0.29^*^** | 2.18 | ± | 0.22 | 2.72 | ± | 0.04 |
| TB (mg/dl) | 0.03 | ± | 0.02 | 0.05 | ± | 0.02 | 0.00 | ± | 0.02 | 0.04 | ± | 0.04 | 0.03 | ± | 0.01 |
| ALP (IU/L) | 655 | ± | 115 | 498 | ± | 55 | 698.2 | ± | 278 | 838 | ± | 353 | 653 | ± | 112 |
| AST (IU/L) | 91 | ± | 4.97 | **387** | **±** | **373^*^** | 124.4 | ± | 51 | 106 | ± | 33 | 84 | ± | 6 |
| ALT (IU/L) | 36 | ± | 21.83 | **137** | **±** | **136^*^** | 28.8 | ± | 10 | 27 | ± | 11 | 23 | ± | 3 |
| γGT (IU/L) | -0.17 | ± | 0.75 | 2.33 | ± | 2.50 | 2.60 | ± | 4.72 | 2.60 | ± | 5.27 | 0.33 | ± | 0.82 |
| Creatinine (mg/dl) | 0.39 | ± | 0.02 | 0.58 | ± | 0.21 | 0.59 | ± | 0.42 | 0.91 | ± | 1.17 | 0.37 | ± | 0.02 |
| Triglyceride (mg/dl) | 28.00 | ± | 10.86 | 19.50 | ± | 4.32 | 30.20 | ± | 23.42 | 38.60 | ± | 31.05 | 37.17 | ± | 12.94 |
| Glucose (mg/L) | 142.17 | ± | 18.02 | 91.50 | ± | 31.10 | 143.00 | ± | 55.10 | 161.80 | ± | 41.99 | 116.00 | ± | 9.86 |
| A/G | 0.90 | ± | 0.11 | 0.80 | ± | 0.15 | **0.70** | **±** | **0.12^**^** | **0.68** | **±** | **0.08^**^** | 0.90 | ± | 0.00 |
| Potassium (mEq/L) | 4.52 | ± | 0.58 | 4.70 | ± | 0.81 | 4.94 | ± | 0.77 | 5.46 | ± | 2.52 | 4.60 | ± | 0.32 |
| Chlorine (mEq/L) | 101.83 | ± | 1.83 | 102.00 | ± | 0.89 | 103.20 | ± | 2.05 | 101.00 | ± | 1.58 | 100.17 | ± | 0.75 |
| Sodium (mEq/L) | 143.83 | ± | 0.98 | 144.83 | ± | 1.17 | 144.20 | ± | 3.49 | 142.60 | ± | 2.70 | 143.67 | ± | 0.82 |
| Calcium (mg/dl) | 9.63 | ± | 0.21 | 9.55 | ± | 0.16 | 9.54 | ± | 0.45 | 9.86 | ± | 0.29 | 9.62 | ± | 0.12 |
| Phosphorus (mg/dl) | 6.50 | ± | 0.37 | 7.72 | ± | 0.53 | 8.24 | ± | 1.19 | **9.30** | **±** | **3.11^*^** | 7.65 | ± | 0.55 |
| ***n (female)*** | *6* | | | *6* | | | *6* | | | *6* | | | *6* | | |
| BUN (mg/dl) | 18.67 | ± | 1.92 | **24.25** | **±** | **5.26^*^** | 17.30 | ± | 2.71 | 19.12 | ± | 3.69 | 18.62 | ± | 1.47 |
| TC (mg/dl) | 74.33 | ± | 9.81 | **57.83** | **±** | **12.38^*^** | **56.80** | **±** | **6.87^*^** | **49.00** | **±** | **12.02^*^** | **54.33** | **±** | **7.69^*^** |
| Total Protein (g/dl) | 4.00 | ± | 0.63 | 6.33 | ± | 4.80 | 6.00 | ± | 3.94 | 3.60 | ± | 0.89 | 3.17 | ± | 0.98 |
| LDL (mg/dl) | 24.83 | ± | 1.94 | **17.83** | **±** | **6.46^*^** | 18.60 | ± | 4.22 | 19.20 | ± | 3.96 | 21.00 | ± | 2.53 |
| HDL (mg/dl) | 5.47 | ± | 0.12 | 5.45 | ± | 0.16 | 5.18 | ± | 0.29 | 5.48 | ± | 0.19 | 5.33 | ± | 0.21 |
| Albumin (g/dl) | 2.70 | ± | 0.09 | 2.62 | ± | 0.12 | **2.24** | **±** | **0.36^*^** | 2.68 | ± | 0.08 | 2.77 | ± | 0.08 |
| TB (mg/dl) | 0.00 | ± | 0.03 | 0.20 | ± | 0.37 | 0.01 | ± | 0.01 | 0.02 | ± | 0.04 | 0.01 | ± | 0.01 |
| ALP (IU/L) | 475 | ± | 44 | 481 | ± | 64 | 543 | ± | 82 | 475 | ± | 65 | 517 | ± | 36 |
| AST (IU/L) | 112 | ± | 5 | 334 | ± | 419 | 118 | ± | 45 | 140 | ± | 42 | 99 | ± | 9 |
| ALT (IU/L) | 24 | ± | 3 | **84** | **±** | **56^*^** | 27 | ± | 2 | 42 | ± | 7 | 27. | ± | 2 |
| γGT (IU/L) | 0.33 | ± | 0.82 | 1.17 | ± | 1.94 | 0.40 | ± | 0.55 | 0.20 | ± | 0.45 | 0.00 | ± | 0.00 |
| Creatinine (mg/dl) | 0.41 | ± | 0.04 | 0.44 | ± | 0.01 | 0.42 | ± | 0.04 | 0.43 | ± | 0.06 | 0.41 | ± | 0.03 |
| Triglyceride (mg/dl) | 28.00 | ± | 11.44 | 52.00 | ± | 85.37 | 18.40 | ± | 9.86 | 14.80 | ± | 10.85 | 22.67 | ± | 12.34 |
| Glucose (mg/L) | 83.00 | ± | 17.98 | 55.33 | ± | 9.97 | 101.00 | ± | 56.05 | 80.80 | ± | 30.88 | 65.83 | ± | 9.17 |
| A/G | 0.98 | ± | 0.08 | 0.92 | ± | 0.04 | **0.78** | **±** | **0.16^*^** | 0.96 | ± | 0.05 | 1.07 | ± | 0.05 |
| Potassium (mEq/L) | 4.50 | ± | 0.35 | 4.22 | ± | 0.17 | 4.72 | ± | 0.37 | 4.46 | ± | 0.67 | 4.42 | ± | 0.40 |
| Chlorine (mEq/L) | 100.50 | ± | 2.88 | 101.67 | ± | 1.03 | 102.40 | ± | 1.95 | 102.80 | ± | 2.68 | 103.50 | ± | 1.22 |
| Sodium (mEq/L) | 142.33 | ± | 3.20 | 145.50 | ± | 1.38 | 145.20 | ± | 2.68 | 145.40 | ± | 1.34 | 144.17 | ± | 0.98 |
| Calcium (mg/dl) | 9.77 | ± | 0.29 | 9.73 | ± | 0.37 | 9.48 | ± | 0.33 | 9.66 | ± | 0.21 | 9.47 | ± | 0.08 |
| Phosphorus (mg/dl) | 7.65 | ± | 0.71 | 7.58 | ± | 0.93 | 7.76 | ± | 0.64 | 7.96 | ± | 0.54 | 7.25 | ± | 0.38 |
|  |  |  |  |  |  |  |  |  |  |  |  |  |  | | |

**Supplementary Table 2.** Serum biochemistry values for NALM-6 xenograft rats injected with CD19 CAR-T cells

*, *p* < 0.05, **, *p* < 0.01.

**Supplementary Table 3.** Organ weight of male NALM-6 xenograft rats injected with CD19 CAR-T cells

| male | | No tumor | Tumor only | Mock T | CAR-T Low | CAR-T High |
| --- | --- | --- | --- | --- | --- | --- |
| Liver | (g) | 6.107 ± 1.379 | 12.697 ± 4.282 | **15.264 ± 10.921*** | 9.413 ± 3.279 | 5.764 ± 0.895 |
|  | (%BW) | 3.205 ± 0.360 | **9.545 ± 4.282*** | **10.726 ± 6.699**** | 5.677 ± 1.259 | 3.071 ± 0.095 |
| Spleen | (g) | 0.248 ± 0.038 | 0.394 ± 0.209 | 0.405 ± 0.157 | 0.393 ± 0.223 | 0.231 ± 0.036 |
|  | (%BW) | 0.132 ± 0.015 | **0.284 ± 0.115*** | **0.295 ± 0.137*** | 0.261 ± 0.094 | 0.123 ±0.007 |
| Kidney (right) | (g) | 0.808 ± 0.140 | 1.289 ± 0.554 | 1.499 ± 1.330 | 0.961 ± 0.152 | 0.809 ± 0.107 |
|  | (%BW) | 0.430 ± 0.042 | 0.946 ± 0.372 | 1.1315 ± 1.017 | 0.632 ± 0.273 | 0.432 ± 0.011 |
| Kidney (left) | (g) | 0.775 ± 0.142 | 1.329 ± 1.137 | 1.036 ± 0.498 | 1.501 ± 1.193 | 0.796 ± 0.116 |
|  | (%BW) | 0.411 ± 0.030 | 0.971 ± 0.723 | 0.762 ± 0.420 | 0.923 ± 0.665 | 0.425 ± 0.026 |
| Adrenal gland (right) | (g) | 0.018 ± 0.001 | 0.021 ± 0.005 | **0.025 ± 0.004**** | 0.023 ± 0.004 | 0.019 ± 0.003 |
|  | (%BW) | 0.010 ± 0.002 | **0.015 ± 0.002*** | **0.019 ± 0.006***** | 0.015 ± 0.003 | 0.010 ± 0.001 |
| Adrenal gland (left) | (g) | 0.019 ± 0.003 | 0.021 ± 0.005 | **0.031 ± 0.008**** | 0.023 ± 0.003 | 0.019 ± 0.003 |
|  | (%BW) | 0.010 ± 0.002 | 0.015 ± 0002 | **0.022 ± 0.007***** | 0.014 ± 0.003 | 0.010 ± 0.001 |
| Testis (right) | (g) | 1.270 ± 0.149 | **0.928 ± 0.171**** | 1.046 ± 0.142 | 1.192 ± 0.143 | 1.322 ± 0.140 |
|  | (%BW) | 0.682 ± 0.087 | 0.710 ± 0.166 | 0.753 ± 0.130 | 0.752 ± 0.144 | 0.709 ± 0.041 |
| Testis (left) | (g) | 1.301 ± 0.154 | 0.956 ± 0.164 | 1.064 ± 0.164 | 1.239 ± 0.212 | 1.333 ± 0.102 |
|  | (%BW) | 0.698 ± 0.087 | 0.741 ± 0.217 | 0.761 ± 0.114 | 0.785 ± 0.191 | 0.719 ± 0.067 |
| Heart | (g) | 0.706 ± 0.098 | **0.500 ± 0.082**** | 0.696 ± 0.110 | 0.643 ± 0.089 | 0.660 ± 0.076 |
|  | (%BW) | 0.378 ± 0.042 | 0.386 ± 0.100 | **0.501 ± 0.096*** | 0.401 ± 0.040 | 0.354 ± 0.028 |
| Lung | (g) | 0.883 ± 0.121 | 0.795 ± 0.099 | 1.020 ± 0.331 | 0.887 ± 0.095 | 0.802 ± 0.093 |
|  | (%BW) | 0.446 ± 0.052 | 0.619 ± 0.184 | **0.732 ± 0.211**** | 0.557 ± 0.078 | 0.430 ± 0.029 |
| Brain | (g) | 1.786 ± 0.075 | 1.671 ± 0.145 | 1.781 ± 0.120 | 1.804 ± 0.032 | 1.778 ± 0.059 |
|  | (%BW) | 0.997 ± 0.229 | 1.315 ± 0.431 | 1.298 ± 0.272 | 1.151 ± 0.275 | 0.966 ± 0.146 |

*, *p* < 0.05, **, *p* < 0.01.

**Supplementary Table 4.** Organ weight of female NALM-6 xenograft rats injected with CD19 CAR-T cells

| female | | No tumor | Tumor only | Mock T | CAR-T Low | CAR-T High |
| --- | --- | --- | --- | --- | --- | --- |
| Liver | (g) | 3.667 ± 0.562 | **10.017 ± 6.062*** | 7.013 ± 3.175 | 7.272 ± 4.697 | 3.405 ± 0.389 |
|  | (%BW) | 2.965 ± 0.148 | 8.528 ± 5.385 | 6.668 ± 3.931 | 6.761 ± 5.229 | 2.991 ± 0.138 |
| Spleen | (g) | 0.180 ± 0.052 | 0.175 ± 0.050 | 0.226 ± 0.125 | 0.207 ± 0.080 | 0.141 ± 0.038 |
|  | (%BW) | 0.144 ± 0.027 | 0.145 ± 0.031 | 0.215 ± 0.149 | 0.188 ± 0.088 | 0.123 ± 0.026 |
| Kidney (right) | (g) | 0.510 ± 0.066 | 0.550 ± 0.010 | 0.632 ± 0.082 | 0.620 ± 0.220 | 0.532 ± 0.037 |
|  | (%BW) | 0.414 ± 0.024 | 0.465 ± 0.041 | 0.582 ± 0.129 | 0.570 ± 0.270 | 0.470 ± 0.037 |
| Kidney (left) | (g) | 0.514 ± 0.065 | 0.486 ± 0.047 | 0.658 ± 0.110 | 0.639 ± 0.234 | 0.528 ± 0.032 |
|  | (%BW) | 0.417 ± 0.023 | 0.023 ± 0.001 | 0.602 ± 0.127 | 0.582 ± 0.283 | 0.467 ± 0.045 |
| Adrenal gland (right) | (g) | 0.018 ± 0.009 | 0.023 ± 0.019 | 0.053 ± 0.063 | 0.020 ± 0.004 | 0.020 ± 0.002 |
|  | (%BW) | 0.014 ± 0.006 | 0.019 ± 0.002 | 0.053 ± 0.071 | 0.018 ± 0.004 | 0.017 ± 0.002 |
| Adrenal gland (left) | (g) | 0.018 ± 0.005 | 0.027 ± 0.008 | 0.028 ± 0.008 | 0.021 ± 0.004 | 0.021 ± 0.001 |
|  | (%BW) | 0.015 ± 0.006 | 0.023 ± 0.008 | **0.025 ± 0.008*** | 0.018 ± 0.004 | 0.018 ± 0.002 |
| Ovary (right) | (g) | 0.025 ± 0.007 | 0.017 ± 0.013 | 0.020 ± 0.014 | 0.029 ± 0.009 | 0.020 ± 0.004 |
|  | (%BW) | 0.020 ± 0.003 | 0.014 ± 0.012 | 0.017 ± 0.011 | 0.026 ± 0.010 | 0.018 ± 0.004 |
| Ovary (left) | (g) | 0.023 ± 0.006 | 0.022 ± 0.00 | 0.031 ± 0.013 | 0.024 ± 0.008 | 0.018 ± 0.005 |
|  | (%BW) | 0.019 ± 0.004 | 0.018 ± 0.004 | 0.028 ± 0.010 | 0.021 ± 0.008 | 0.016 ± 0.004 |
| Heart | (g) | 0.460 ± 0.083 | 0.510 ± 0.107 | 0.429 ± 0.059 | 0.460 ± 0.087 | 0.479 ± 0.068 |
|  | (%BW) | 0.372 ± 0.035 | 0.425 ± 0.082 | 0.388 ± 0.035 | 0.403 ± 0.044 | 0.421 ± 0.043 |
| Lung | (g) | 0.634 ± 0.069 | 0.611 ± 0.128 | 0.767 ± 0.158 | 0.668 ± 0.052 | 0.625 ± 0.038 |
|  | (%BW) | 0.515 ± 0.034 | 0.511 ± 0.112 | **0.713 ± 0.235*** | 0.594 ± 0.062 | 0.552 ± 0.030 |
| Brain | (g) | 1.651 ± 0.073 | 1.673 ± 0.022 | 1.673 ± 0.064 | 1.617 ± 0.062 | 1.666 ± 0.046 |
|  | (%BW) | 1.350 ± 0.123 | 1.400 ± 0.086 | 1.528 ± 0.181 | 1.438 ± 0.142 | 1.473 ± 0.120 |

*, *p* < 0.05, **, *p* < 0.01.

**Supplementary Table 5.** Results of microscopic observation of NALM-6 xenograft rats injected with CD19 CAR-T cells

|  |  | **Male** | | | | | | | | |  | **Female** | | | | | | | | |
| --- | --- | --- | --- | --- | --- | --- | --- | --- | --- | --- | --- | --- | --- | --- | --- | --- | --- | --- | --- | --- |
|  |  | No tumor | | Tumor only | | Mock T | | CAR-T  Low | | CAR-T  High |  | No tumor | | Tumor only | Mock T | | CAR-T  Low | | CAR-T  High | |
| Liver | Normal | 6/6 | | 0/6 | | 0/6 | | 0/6 | | 6/6 |  | 6/6 | | 2/6 | 3/6 | | 1/6 | | 6/6 | |
|  | Tumor cell infiltration | 0/6 | | **6/6** | | **6/6** | | **6/6** | | 0/6 |  | 0/6 | | **4/6** | **3/6** | | **5/6** | | 0/6 | |
| Spleen | Normal | 6/6 | | 5/6 | | 5/6 | | 4/6 | | 6/6 |  | 6/6 | | 5/6 | 5/6 | | 5/6 | | 6/6 | |
|  | Tumor cell infiltration | 0/6 | | **1/6** | | **1/6** | | 2/6 | | 0/6 |  | 0/6 | | **1/6** | **1/6** | | **1/6** | | 0/6 | |
| Kidney | Normal | 6/6 | | 0/6 | | 0/6 | | 0/6 | | 0/6 |  | 6/6 | | 0/6 | 0/6 | | 1/6 | | 6/6 | |
|  | Tumor cell infiltration | 0/6 | | **6/6** | | **6/6** | | **6/6** | | 0/6 |  | 0/6 | | **6/6** | **6/6** | | **5/6** | | 0/6 | |
| Pancreas | Normal | 6/6 | | 3/6 | | 1/6 | | 2/6 | | 6/6 |  | 6/6 | | 2/6 | 0/6 | | 6/6 | | 6/6 | |
|  | Tumor cell infiltration | 0/6 | | **1/6** | | 0/6 | | 0/6 | | 0/6 |  | 0/6 | | 0/6 | 0/6 | | 0/6 | | 0/6 | |
|  | Acinar cell degranulation | 0/6 | | **1/6** | | **1/6** | | 0/6 | | 0/6 |  | 0/6 | | **1/6** | **3/6** | | 0/6 | | 0/6 | |
|  | Peritonitis | 0/6 | | **1/6** | | **4/6** | | **4/6** | | 0/6 |  | 0/6 | | **3/6** | **3/6** | | 0/6 | | 0/6 | |
| Adrenal  gland | Normal | 6/6 | | 4/6 | | 1/6 | | 3/6 | | 6/6 |  | 6/6 | | 3/6 | 4/6 | | 6/6 | | 6/6 | |
|  | Tumor cell infiltration | 0/6 | | **2/6** | | **2/6** | | **2/6** | | 0/6 |  | 0/6 | | **3/6** | **1/6** | | 0/6 | | 0/6 | |
|  | Peritonitis | 0/6 | | 0/6 | | **3/6** | | **1/6** | | 0/6 |  | 0/6 | | 0/6 | 0/6 | | 0/6 | | 0/6 | |
|  | Cortex necrosis | 0/6 | | 0/6 | | 0/6 | | 0/6 | | 0/6 |  | 0/6 | | 0/6 | **1/6** | | 0/6 | | 0/6 | |
| Lung | Normal | 6/6 | | 3/6 | | 6/6 | | 2/6 | | 5/6 |  | 6/6 | | 5/6 | 5/6 | | 5/6 | | 6/6 | |
|  | Tumor cell infiltration | 0/6 | | **2/6** | | 0/6 | | **2/6** | | **1/6** |  | 0/6 | | **1/6** | **1/6** | | **1/6** | | 0/6 | |
|  | Inflammatory cell infiltration | 0/6 | | **1/6** | | 0/6 | | **2/6** | | 0/6 |  | 0/6 | | 0/6 | 0/6 | | 0/6 | | 0/6 | |
| Brain | Normal | 6/6 | | 5/6 | | 5/6 | | 4/6 | | 6/6 |  | 6/6 | | 6/6 | 5/6 | | 6/6 | | 6/6 | |
|  | Tumor cell infiltration | 0/6 | | **1/6** | | **1/6** | | **2/6** | | 0/6 |  | 0/6 | | 0/6 | 0/6 | | 0/6 | | 0/6 | |
|  | Neutrophilic infiltration | 0/6 | | 0/6 | | 0/6 | | 0/6 | | 0/6 |  | 0/6 | | 0/6 | **1/6** | | 0/6 | | 0/6 | |
| Femur | Normal | 6/6 | | 2/6 | | 3/6 | | 4/6 | | 6/6 |  | 6/6 | | 5/6 | 4/6 | | 4/6 | | 6/6 | |
|  | Tumor cell infiltration | 0/6 | | **4/6** | | **3/6** | | **2/6** | | 0/6 |  | 0/6 | | **1/6** | **2/6** | | **2/6** | | 0/6 | |
| Heart | Normal | 6/6 | | 5/6 | | 2/6 | | 3/6 | | 6/6 |  | 6/6 | | 6/6 | 3/6 | | 6/6 | | 6/6 | |
|  | Tumor cell infiltration | 0/6 | | 0/6 | | **1/6** | | 0/6 | | 0/6 |  | 0/6 | | 0/6 | **1/6** | | 0/6 | | 0/6 | |
|  | Inflammatory cell infiltration | 0/6 | | **1/6** | | **3/6** | | **3/6** | | 0/6 |  | 0/6 | | 0/6 | **2/6** | | 0/6 | | 0/6 | |
| Sternum | Normal | 6/6 | | 0/6 | | 0/6 | | 2/6 | | 6/6 |  | 6/6 | | 2/6 | 2/6 | | 6/6 | | 6/6 | |
|  | Tumor cell infiltration | 0/6 | | **6/6** | | **6/6** | | **3/6** | | 0/6 |  | 0/6 | | **4/6** | **4/6** | | 0/6 | | 0/6 | |
| Spinal Cord | Normal | 6/6 | | 2/6 | | 3/6 | | 3/6 | | 6/6 |  | 6/6 | | 4/6 | 4/6 | | 6/6 | | 6/6 | |
|  | Tumor cell infiltration | 0/6 | | **4/6** | | **3/6** | | **3/6** | | 0/6 |  | 0/6 | | **2/6** | **2/6** | | 0/6 | | 0/6 | |
| Nasal cavity | Normal | 6/6 | | 4/6 | | 4/6 | | 3/6 | | 6/6 |  | 6/6 | | 5/6 | 5/6 | | 6/6 | | 6/6 | |
|  | Tumor cell infiltration | 0/6 | | **2/6** | | **2/6** | | **3/6** | | 0/6 |  | 0/6 | | **1/6** | **1/6** | | 0/6 | | 0/6 | |
|  |  |  |  | |  | |  | |  | |  |  |  | | |  | |  | |  |
|  |  | **Male** | | | | | | | | |  | **Female** | | | | | | | | |
|  |  | No tumor | | Tumor only | | Mock T | | CAR-T  Low | | CAR-T  High |  | No tumor | | Tumor only | Mock T | | CAR-T  Low | | CAR-T  High | |
| Stomach | Normal | 6/6 | | 2/6 | | 0/6 | | 3/6 | | 5/6 |  | 6/6 | | 0/6 | 3/6 | | 3/6 | | 5/6 | |
|  | Erosion | 0/6 | | **2/6** | | **4/6** | | 0/6 | | 0/6 |  | 0/6 | | **1/6** | **2/6** | | 0/6 | | 0/6 | |
|  | Edema | 0/6 | | **2/6** | | 0/6 | | **1/6** | | 0/6 |  | 0/6 | | **1/6** | **1/6** | | 0/6 | | 0/6 | |
|  | Apoptosis |  | |  | |  | |  | |  |  | 0/6 | |  |  | |  | |  | |
|  | ....*minimal* | 0/6 | | 0/6 | | **1/6** | | **2/6** | | **1/6** |  | 0/6 | | **3/6** | 0/6 | | **3/6** | | **1/6** | |
|  | ........s*light* | 0/6 | | 0/6 | | **1/6** | | 0/6 | | 0/6 |  | 0/6 | | **1/6** | 0/6 | | 0/6 | | 0/6 | |
| Duodenum | Normal | 6/6 | | 6/6 | | 6/6 | | 6/6 | | 6/6 |  | 6/6 | | 6/6 | 6/6 | | 6/6 | | 6/6 | |
| Jejunum | Normal | 6/6 | | 6/6 | | 4/6 | | 3/6 | | 6/6 |  | 6/6 | | 6/6 | 6/6 | | 6/6 | | 6/6 | |
|  | Peritonitis | 0/6 | | 0/6 | | **2/6** | | **3/6** | | 0/6 |  | 0/6 | | 0/6 | 0/6 | | 0/6 | | 0/6 | |
| Ileum | Normal | 6/6 | | 6/6 | | 4/6 | | 3/6 | | 6/6 |  | 6/6 | | 6/6 | 6/6 | | 6/6 | | 6/6 | |
|  | Peritonitis | 0/6 | | 0/6 | | **2/6** | | **3/6** | | 0/6 |  | 0/6 | | 0/6 | 0/6 | | 0/6 | | 0/6 | |
| Colon/ Rectum | Normal | 6/6 | | 6/6 | | 2/6 | | 2/6 | | 6/6 |  | 6/6 | | 5/6 | 4/6 | | 3/6 | | 6/6 | |
|  | Peritonitis | 0/6 | | 0/6 | | **4/6** | | **4/6** | | 0/6 |  | 0/6 | | **1/6** | **2/6** | | 0/6 | | 0/6 | |
|  | Apoptosis | 0/6 | | 0/6 | | 0/6 | | 0/6 | | 0/6 |  | 0/6 | | 0/6 | 0/6 | | **3/6** | | 0/6 | |
| Thyroid gland/ | Normal | 6/6 | | 6/6 | | 6/6 | | 6/6 | | 6/6 |  | 6/6 | | 6/6 | 6/6 | | 6/6 | | 6/6 | |
| Parathyroid gland | Normal | 6/6 | | 6/6 | | 6/6 | | 6/6 | | 6/6 |  | 6/6 | | 6/6 | 6/6 | | 6/6 | | 6/6 | |
| Urinary bladder | Normal | 6/6 | | 6/6 | | 6/6 | | 6/6 | | 6/6 |  | 6/6 | | 6/6 | 6/6 | | 6/6 | | 6/6 | |
| Salivary gland | Normal | 6/6 | | 6/6 | | 6/6 | | 6/6 | | 6/6 |  | 6/6 | | 6/6 | 6/6 | | 6/6 | | 6/6 | |
| Testis | Normal | 6/6 | | 6/6 | | 6/6 | | 6/6 | | 6/6 |  | - | | - | - | | - | | - | |
| Epididymis | Normal | 6/6 | | 6/6 | | 6/6 | | 6/6 | | 6/6 |  | - | | - | - | | - | | - | |
| Prostate | Normal | 6/6 | | 6/6 | | 6/6 | | 6/6 | | 6/6 |  | - | | - | - | | - | | - | |
| Ovary/ Uterus | Normal | - | | - | | - | | - | | - |  | 6/6 | | 6/6 | 6/6 | | 6/6 | | 6/6 | |
| Eye | Normal | 6/6 | | 6/6 | | 6/6 | | 6/6 | | 6/6 |  | 6/6 | | 6/6 | 6/6 | | 6/6 | | 6/6 | |
| Harderian gland | Normal | 6/6 | | 6/6 | | 6/6 | | 6/6 | | 6/6 |  | 6/6 | | 6/6 | 6/6 | | 6/6 | | 6/6 | |
| Skin | Normal | 6/6 | | 6/6 | | 6/6 | | 6/6 | | 6/6 |  | 6/6 | | 6/6 | 6/6 | | 6/6 | | 6/6 | |
| Pituitary gland | Normal | 6/6 | | 6/6 | | 6/6 | | 6/6 | | 6/6 |  | 6/6 | | 6/6 | 6/6 | | 6/6 | | 6/6 | |
|  |  |  |  | |  | |  | |  | |  |  |  | | |  | |  | |  |
